# Methyltransferase-like 3 (METTL3) inhibition potentiates anti-tumor immunity: a novel strategy for improving anti-PD1 therapy

**DOI:** 10.1038/s41392-023-01696-x

**Published:** 2023-12-11

**Authors:** Anna Brichkina, Roman Suezov, Magdalena Huber

**Affiliations:** Institute of Systems Immunology, Member of the German Center for Lung Research (DZL), Philipps University of Marburg, 35043 Marburg, Germany

**Keywords:** Tumour immunology, Cancer therapy

In a recent article published in *Cancer Discovery*, Guirguis et al. demonstrated that enzymatic inhibitors of the m^6^A methyltransferase METTL3 induced a formation of immunostimulatory double-stranded mRNAs (dsRNAs) in cancer cells, which triggered a profound tumor-cell-intrinsic interferon response leading to an enhanced antigen presentation and thereby increased killing of tumor cells by cytotoxic CD8^+^ T-lymphocytes. The inhibition of METTL3 synergized with anti-PD1 immunotherapy, highlighting its potential for improving cancer treatment.^1^

The paradigm in cancer treatment has shifted towards T-cell immunotherapy, because of specific, antigen-directed cytotoxicity and long-term memory against tumor cells. The effectiveness of T-cell cytotoxicity often hinges on the immunogenicity of tumors, which employ various mechanisms to evade immune attack. This includes impairing the presentation of antigens through downregulating major histocompatibility complex I (MHC-I). Furthermore, tumors express programmed death ligand 1 (PD-L1) to evade anti-tumor immune responses. However, PD-L1 expression also associates with higher T-cell infiltration, suggesting that tumors with increased PD-L1 levels elicit a stronger immune response to anti-PD1 immune checkpoint inhibitor (ICI) therapy. These facts highlight potential benefits of combining ICI therapy with upregulating tumor-specific antigen presentation or other strategies to overcome tumor resistance to immunotherapy.

Recent studies have revealed that mammalian cells can accumulate self-derived dsRNAs when various cellular processes are dysregulated. One mechanism leading to such immunogenic dsRNA structure formation is a defective installation of RNA modifications. N6-methyladenosine (m^6^A), the most abundant RNA modification, significantly influences the stability, translation, and conformation of modified transcripts. The absence of m^6^A leads to the formation of endogenous dsRNAs and consequently to activation of interferon (IFN) pathways similar to viral-infected cells. The m^6^A methyltransferase METTL3 is responsible for creating m^6^A marks. Studies have demonstrated that downregulation of METTL3 in acute myeloid leukemia (AML) cells resulted in cell cycle arrest, differentiation, apoptosis, and delays in leukemia progression in vivo.^2,3^ Furthermore, the loss of METTL3 and m^6^A induces aberrant dsRNA formation in hematopoietic cells, triggering an innate immune response and cell death.^4^ Pharmaceutical inhibitor STM2457, targeting METTL3, has shown remarkable effectiveness in reducing AML growth in vitro and in vivo,^5^ emphasizing enzymatic inhibition of METTL3 as a promising strategy for anticancer therapy. However, the precise molecular mechanisms underlying the antitumor response elicited by METTL3 inhibition remained not fully understood and required further investigation.

**Fig. 1:**
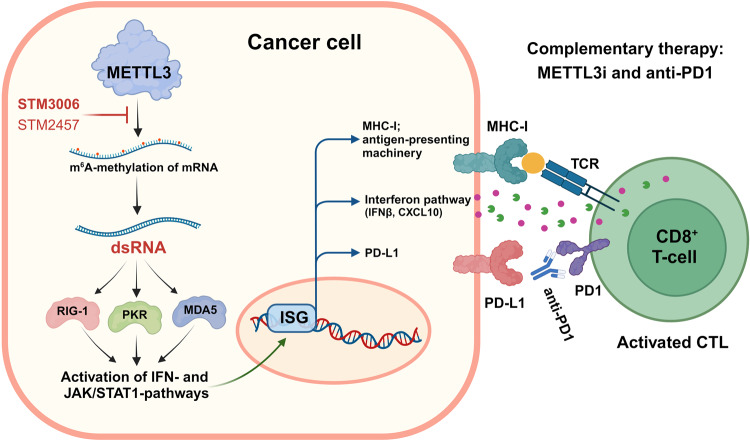
Inhibition of METTL3 induces the formation and detection of dsRNA and potentiates anti-PD1 immunotherapy. Methyltransferase METTL3 marks mRNA with N^6^-Methyladenosine (m^6^A). Enzymatic inhibitors of METTL3 (METTL3i), such as STM2457 or STM3006, efficiently block m^6^A methylation of mRNA, leading to the formation of immunogenic double-stranded mRNAs (dsRNAs). Within cancer cells, intracellular enzymes PKR, MDA5 and RIG-1 sense these dsRNAs, triggering the activation of a cell-intrinsic interferon response (IFN). This process is mediated by the JAK/STAT1 signaling pathway, which is involved in upregulating interferon-stimulating genes (ISGs), surface MHC-I and PD-L1, as well in the secretion of cytokines and chemokines, such as IFNβ and CXCL10. Consequently, cytotoxic T lymphocytes (CTL) kill tumor cells more efficiently. Finally, a combination of METTL3 inhibition and anti-PD1 therapy synergistically improves tumor eradication by cytotoxic CD8^+^ T cells. Created with BioRender.com

The collaborative efforts of the Dawson and Rausch teams resulted in the development and characterization of a highly potent and selective inhibitor of METTL3 known as STM3006.^1^ This inhibitor, structurally distinct from the previously described STM2457,^5^ exhibits significantly improved potency. To gain insight into the transcriptional effects of targeting METTL3 in tumor cells, the authors observed upregulation of IFNβ, interferon-stimulated genes (ISGs) including *OAS2*, *MDA5*, *IFIT1*, *ISG15*, chemokine CXCL10, and surface MHC-I. Mechanistically, both inhibitors induced the formation of immunogenic dsRNA activating ISGs and genes associated with antigen presentation in tumor cells of different origins. Interestingly, the most significant change in m^6^A levels was observed in mRNAs belonging to the antigen presentation machinery, including *H2K1*, *Calr*, and *Pdia3*, but not in transcripts induced by interferon. These findings suggest a more pronounced gene and selective alteration of m^6^A status and thereby stabilization in individual newly synthesized transcripts of the MHC-I pathway upon METTL3 inhibition (Fig. 1). This demonstrates that catalytic inhibition of METTL3 does not considerably impact the transcription process itself, either under steady-state conditions or after stimulation with inflammatory cytokines.

To analyze consequences of upregulated IFN pathway and enhanced antigen presentation resulting from METTL3 inhibition in tumor cells, the authors employed an antigen-specific co-culture system consisting of tumor cells expressing the model antigen ovalbumin (OVA) and OVA-specific transgenic CD8^+^ T-cells (OT-I). Both STM2457 and STM3006 compounds increased killing of cancer cells by activated OT-I T-cells. Importantly, CRISPR screening further confirmed that the effectiveness of METTL3 inhibition in eradicating cancer is heavily reliant on tumor’s intrinsic capability to detect dsRNA through RIG-I, OAS3, RNAse-L, PKR, and IFIH, along with a functional JAK/STAT signaling pathway and an intact MHC-I antigen presentation. Additionally, METTL3 inhibition led to the increased expression of PD-L1 on the surface of tumor cells, suggesting the potential to enhance therapies targeting the PD1/PD-L1 ICI. In vivo experiments showed that STM2457, particularly in combination with anti-PD1 treatment, significantly improved the survival of mice transplanted with breast cancer cells (AT3)-expressing OVA, both with and without adoptive transfer of OT-I T-cells. This highlights the promising potential of short-course treatment with METTL3 inhibitors together with immunotherapy blocking PD1, which could improve disease control. However, not all treated mice achieved a cure. To explore the mechanisms of resistance to therapy, the authors conducted further investigations. Using SPLINTR lineage tracing methodology and single-cell RNAseq of resistant clones, they discovered that anti-PD1 therapy and inhibitors of METTL3 target distinct populations of cancer cells. Crucially, the combination of both treatments was effective in eliminating immune-escaped malignant cells that were resistant to either agent on its own. Notably, the clones insensitive to STM2457 showed downregulation of genes linked to antigen processing and presentation. On the other hand, clones that became irresponsive to the complementary therapy exhibited a combined signature of resistance to each single treatment. Altogether, this research offers both molecular insights and pre-clinical evidence, supporting the use of pharmacological drugs targeting the m^6^A methyltransferase METTL3 as a valuable combination therapy to enhance anti-tumor immune responses and improve the effectiveness of anti-PD1 immune checkpoint blockage therapy (Fig. 1).

In summary, Guirgius et al. discovered a novel mechanism potentiating anti-tumor immune response by inducing dsRNA in cancer cells through pharmacological inhibition of METTL3. Enzymatic inhibitors of METTL3 may be suitable for patients with solid or hematological cancers eligible for anti-PD1 ICI to improve treatment outcomes. However, the study does not exclude the potential effect of METTL3 inhibition on other immune cells, such as macrophages or dendritic cells, which could enhance their activity and antigen presentation through upregulated interferon responses. Overcoming MHC-I downregulation by targeting METTL3, or similar RNA-modifying enzymes, may potentiate other T-cell-based cancer therapies, including CAR-T and adoptive T-cell transfer, and it might also be useful for stimulating T-cell responses in chronic viral infections.
